# Prevalence and determinants of low protein intake in very old adults: insights from the Newcastle 85+ Study

**DOI:** 10.1007/s00394-017-1537-5

**Published:** 2017-09-25

**Authors:** Nuno Mendonça, Antoneta Granic, John C. Mathers, Tom R. Hill, Mario Siervo, Ashley J. Adamson, Carol Jagger

**Affiliations:** 1 School of Agriculture Food and Rural Development, Newcastle University, Newcastle upon Tyne, NE1 7RU UK; 2 Newcastle University Institute for Ageing, Newcastle University, Newcastle upon Tyne, NE2 4AX UK; 3 Human Nutrition Research Centre, Newcastle University, Newcastle upon Tyne, NE2 4HH UK; 4 Institute of Neuroscience, Newcastle University, Newcastle upon Tyne, NE2 4HH UK; 5 NIHR Newcastle Biomedical Research Centre in Ageing and Chronic Disease, Newcastle University and Newcastle upon Tyne NHS Foundation Trust, Newcastle upon Tyne, NE4 5PL UK; 6 Institute of Cellular Medicine, Newcastle University, Newcastle upon Tyne, NE4 5PL UK; 7 Institute of Health and Society, Newcastle University, Newcastle upon Tyne, NE4 5PL UK

**Keywords:** Protein, Malnutrition, Aged, 80 and over, Very old, Newcastle 85+

## Abstract

**Purpose:**

The very old (aged ≥ 85 years), fastest growing age group in most western societies, are at especially high risk of muscle mass and strength loss. The amount, sources and timing of protein intake may play important roles in the aetiology and management of sarcopenia. This study investigated the prevalence and determinants of low protein intake in 722 very old adults participating in the Newcastle 85+ Study.

**Methods:**

Protein intake was estimated with 2 × 24-h multiple pass recalls (24 h-MPR) and contribution (%) of food groups to protein intake was calculated. Low protein intake was defined as intake < 0.8 g of protein per adjusted body weight per day. A backward stepwise multivariate linear regression model was used to explore socioeconomic, health and lifestyle predictors of protein intake.

**Results:**

Twenty-eight percent (*n* = 199) of the community-living very old in the Newcastle 85+ Study had low protein intake. Low protein intake was less likely when participants had a higher percent contribution of meat and meat products to total protein intake (OR 0.97, 95% CI 0.95, 1.00) but more likely with a higher percent contribution of cereal and cereal products and non-alcoholic beverages. Morning eating occasions contributed more to total protein intake in the low than in the adequate protein intake group (*p* < 0.001). Being a woman (*p* < 0.001), having higher energy intake (*p* < 0.001) and higher tooth count (*p* = 0.047) was associated with higher protein intake in adjusted models.

**Conclusion:**

This study provides novel evidence on the prevalence of low protein intake, diurnal protein intake patterns and food group contributors to protein intake in the very old.

**Electronic supplementary material:**

The online version of this article (doi:10.1007/s00394-017-1537-5) contains supplementary material, which is available to authorized users.

## Introduction

The very old (aged ≥ 85 years), the fastest growing age group in most western societies, are at especially high risk of malnutrition, sarcopenia and loss of muscle strength. In the UK, 18% of the very old are at medium or high risk of malnutrition (measured with the Malnutrition Universal Screening Tool) [1] and although hospitalised older adults are at relatively higher risk, most very old malnourished people reside at home [2, 3]. Essential amino acids, especially leucine, directly stimulate myofibrillar muscle synthesis through the mTOR pathway and an adequate protein intake is critical to replace losses from catabolic processes [4]. Albeit multifactorial, protein intake, its sources and timing of intake may play important roles in the aetiology and management of ageing-related muscle and strength loss (sarcopenia). In the Dutch National Food Consumption Survey (DNFCS), up to 10% of community-living older adults (≥ 65 years) did not meet the estimated average requirement (EAR) of 0.7 g of protein per kg of body weight per day (g/kg BW/day) [5] and close to 10% of women > 71 years had protein intakes below the EAR in the National Health and Nutrition Examination Survey (NHANES) 2003–2004 [6]. Protein intakes below the current recommended dietary allowance (RDA) of 0.8 g/kg BW/day have been linked to adverse health outcomes, including physical impairment, muscle loss, dementia and mortality [7–9]. On average, older adults have lower protein intakes than their younger counterparts because of loss of independence, changes in oral health and taste perception and, higher incidence of chronic diseases and disabilities [10]. Disease-related catabolism and inflammation may also increase protein requirements [11, 12] which can be coupled with decreased muscle protein responsiveness to protein intake (anabolic resistance) [11].

Research is lacking in understanding of the prevalence and determinants of low protein intake, principal protein food sources, and eating occasions in very old adults. Therefore, utilizing dietary data from the Newcastle 85+ Study we aimed to describe (a) the prevalence and (b) determinants and factors associated with low and adequate protein intake in the very old.

## Methods

### Newcastle 85+ Study

Details of the Newcastle 85+ Study can be found elsewhere [13–15]. Briefly, this longitudinal population-based study approached all people turning 85 in 2006 (born in 1921) in North East England. The recruited cohort was socio-demographically representative of the general UK population [13]. At baseline (2006/2007), multidimensional health assessment and complete general practice (GP) medical records data were available for 845 participants [14], of whom 722 were living in the community, had complete dietary intake data (without protocol violation), and body weight and height measurements.

### Dietary assessment and food group coding

Dietary intake was assessed by 24 h Multiple Pass Recall (24 h-MPR) on two non-consecutive occasions by trained research nurses and portion sizes were estimated using the “Photographic Atlas of Food Portion Sizes” [16, 17]. Energy and protein intakes were estimated using McCance and Widdowson’s sixth edition food composition tables [18]. Individual foods were coded and allocated to 15 first level food groups: cereals and cereal products (CCP), milk and milk products, eggs and egg dishes, oils and fat spreads, meat and meat products (MMP), fish and fish dishes, vegetables, potatoes, savoury snacks, nuts and seeds, fruit, sugar, preserves and confectionery, non-alcoholic beverages, alcoholic beverages and miscellaneous. One approach to estimate protein inadequacy and base recommendations for protein RDA of 0.8 g/kg BW/day is to use adjusted body weight (aBW) defined as the nearest (ideal) body weight that would put an older adult aged ≥ 71 years into a healthy body mass index (BMI) of 22–27 kg/m^2^ [19, 20]. Low protein intake was defined as an intake < 0.8 g/kg of unadjusted bodyweight per day (g/kg aBW/day) and adequate protein intake as ≥ 0.8 g/kg aBW/day. Eating occasions were categorised into 5:30–8:29, 8:30–11:29, 11:30–14:29, 14:30–17:29, 17:30–20:29, 20:30–23:29 and 23:30–5:29. Protein intake distribution was calculated as the coefficient of variation (CV) (standard deviation/total protein intake) between eating occasions (occasions of food intake where energy intake was zero were not included). Higher CVs reflect more skewed protein intake across the day. We also calculated the number of eating occasions that had 20 and 30 g of protein as well as how many participants had at least one of those eating occasions. Misreporting was taken into consideration but not included in the analyses. Briefly, under-reporters and over-reporters were defined as having an EI:BMRest below 1.05 and over 2.0, respectively [21].

### Socioeconomic, lifestyle and health factors

Body weight was adjusted to reflect a healthy body mass index (BMI) in older adults of 22–27 kg/m^2^ and calculated as described in Berner et al. [20]. Briefly, if necessary, the actual body weight was adjusted to the nearest (ideal) weight that would put an individual into an age-appropriate (≥ 71 years) healthy BMI range associated with a decreased risk of mortality. Fat mass and fat-free mass were assessed using a Tanita-305 body-fat analyser (Tanita Corp., Tokyo, Japan). Participants were categorised into the National Statistics Socio-Economic Classification (NS-SEC) three-class scheme [22] based on past main occupation; and, for physical activity, into those with low (scores 0–1), medium (scores 2–6) and high (scores 7–18) based on a purpose designed and validated physical activity questionnaire [23]. Meal provision included meals provided by the social services, voluntary services or other private help in the previous 4 weeks, and luncheon club attendance was defined as at least one visit in the previous 4 weeks. Biomarkers frequently used in malnutrition assessments and included in these analyses are: serum albumin (measured by an automated version of the bromocresol green method), total cholesterol (determined by the cholesterol oxidase/peroxidase method with a Beckman Coulter AU2700 analyser), and high sensitivity C-reactive protein (hs-CRP) (measured with a Dade Behring Cardiophase hsCRP immunoassay). Disease count was created by scoring 17 chronic diseases as either present or absent [24]; a participant with an estimated glomerular filtration rate < 30 ml/min/1.73 m^2^ was defined as renally impaired; global cognition was assessed with the standardised mini-mental state examination (SMMSE; 0–30 points, < 26 indicating cognitive impairment); depression was assessed by the 15-item Geriatric Depression Scale (GDS; ≤ 8 points indicating severe depression); and oral health included swallowing problems (dry mouth and difficulty swallowing for other reasons) and tooth count.

### Statistical analysis

Statistical analysis was conducted using the IBM statistical tool SPSS v22.0. For continuous variables, normality was tested using the Shapiro–Wilk test and confirmed with Q–Q plots; normally distributed data are presented as means and standard deviations (SD), and non-Gaussian distributed variables as medians and interquartile ranges (IQR). Categorical data are presented as percentages (with corresponding sample size). Differences between the low protein intake (< 0.8 g/kg aBW/day) and adequate protein intake (≥ 0.8 g/kg aBW/day) strata were assessed with two sample *t* test and Mann–Whitney *U* test for continuous normally and non-normally distributed variables, respectively, and Chi-squared test (*χ* ^2^) for categorical variables. Sex (men/women), past occupation (routine and manual/intermediate/higher managerial and administrative and professional occupations), education (< 9, 10–11 and ≥ 12 years of full-time education), living alone (no/yes), meal provision (no/yes), luncheon club attendance (no/yes), smoker (no/yes), alcohol drinker (no/yes), physical activity (low, medium, high), diet changed past year (no/yes), energy intake, serum albumin, total cholesterol, hs-CRP, disease count (0–1, 2 and ≥ 3 diseases), renal impairment (yes/no), number of medications, SMMSE, depression (absent, mild, severe), tooth count, swallowing problems (no/yes), self-rated health (fair or poor/good, very good or excellent), able to cook a hot meal and able to go shopping for groceries (no difficulty, able to but with help or an aid, and unable to do this by himself/herself) were entered into a backward stepwise multivariate linear regression model. *p* < 0.05 was considered statistically significant unless otherwise mentioned.

## Results

### Participants’ characteristics by low or high protein intake

The median protein intake of the community-living very old in the Newcastle 85+ Study was 0.97 (0.77–1.24) g/kg aBW/d and 0.99 (0.77–1.24) g/kg unadjusted BW/d. Twenty-eight percent (*n* = 199) of the community-living very old in the Newcastle 85+ Study had a low protein intake (< 0.8 g of protein per kg of aBW) (Table 1 and Supplemental Fig. 1), or 10 fewer people (*n* = 189) if the calculations used unadjusted rather than adjusted body weight. Fifty-four percent (*n* = 390) had protein intakes < 1.0 g/kg aBW/day and 75% (*n* = 539) < 1.2 g/kg aBW/day (Supplemental Fig. 1). Participants with a higher BMI, more fat mass, less energy intake, more even protein distribution throughout the day and lower tooth count had low protein intake (unadjusted models). Albumin and hs-CRP concentrations tended to be lower (*p* = 0.059) and higher (*p* = 0.056), respectively, in the low compared with the adequate protein intake group (≥ 0.8 g/kg aBW/day) (Table 1).

**Table 1:** Health and sociodemographic characteristics of the Newcastle 85+ Study participants with low and adequate protein intakes

	Low protein^a^ (*n* = 199)	Adequate protein^b^ (*n* = 523)	*p* value*
Women (%, *n*)	67.3 (134)	57.2 (299)	0.013
Education (≥ 12 y) (%, *n*)	10.1 (20)	13.5 (70)	0.459
Past occupation (routine and manual) (%, *n*)	52.4 (100)	49.5 (249)	0.625
Living alone (%, *n*)	59.3 (118)	57.3 (299)	0.624
Meal provision (%, *n*)	4.6 (9)	7.2 (36)	0.209
Luncheon club (%, *n*)	7.5 (15)	7.0 (37)	0.818
Unable to prepare hot meal (%, *n*)	9.5 (19)	9.6 (50)	0.692
Unable to go shopping alone (%, *n*)	36.2 (72)	34.4 (180)	0.568
Anthropometry			
BMI (kg/m^2^) (mean, SD)	25.9 (4.7)	24.0 (4.1)	< 0.001
Adjusted body weight (kg)	65.7 (60.2–69.9)	62.4 (56.6–68.8)	0.001
Weight loss (≥ 5% in 3 y) (%, *n*)	47.0 (47)	37.7 (112)	0.101
Fat mass (kg) (mean, SD)	21.7 (8.1)	17.9 (7.3)	< 0.001
Fat-free mass (kg)	43.7 (39.2–52.2)	43.2 (37.5–51.4)	0.136
Lifestyle			
Physical activity (high) (%, *n*)	31.7 (63)	39.5 (206)	0.121
Smokers (%, *n*)	5.6 (11)	6.1 (32)	0.776
Alcohol drinkers (%, *n*)	71.8 (102)	74.1 (269)	0.603
Dietary intake			
Diet changed past year (%, *n*)	7.6 (15)	6.4 (33)	0.564
Total energy (MJ/day)	5.3 (4.2–6.3)	7.4 (6.3–8.8)	< 0.001
Carbohydrates (g/day)	151 (119–192)	209 (172–253)	< 0.001
Total energy from carbohydrates (%)	50.2 (45.1–55.9)	47.9 (43.3–53.4)	< 0.001
Fat (g/day)	48 (37–64)	72 (57–91)	< 0.001
Total energy from fat (%)	35.7 (29.6–40.7)	35.5 (31.2–41.1)	0.084
Total protein (g/day)	42 (37–49)	68 (58–82)	< 0.001
Total energy from protein (%)	13.0 (11.6–16.2)	15.8 (13.8–18.2)	< 0.001
Total protein (g/kg aBW/day)	0.7 (0.6–0.7)	1.1 (0.9–1.3)	< 0.001
Protein distribution (CV) (mean, SD)	0.18 (0.07)	0.19 (0.06)	0.024
Biochemical			
Albumin (g/L)	40 (38–42)	41 (39–42)	0.059
hs-CRP (mg/L)	2.8 (1.4–6.2)	2.4 (1.1–5.7)	0.056
Total cholesterol (mmol/L)	4.8 (3.9–5.8)	4.8 (4.0–5.7)	0.399
Health			
Chronic disease count (≥ 3) (%, *n*)	45.2 (90)	37.7 (197)	0.138
Number of medications	6 (4–9)	6 (3–8)	0.381
Renal impairment (%, *n*)	27.6 (53)	22.1 (112)	0.125
Cognitive impairment (SMMSE < 26) (%, *n*)	21.5 (53)	26.6 (112)	0.139
Severe depression (%, *n*)	7.1 (14)	7.3 (37)	0.907
Swallowing problems (%, *n*)	56.3 (112)	58.2 (304)	0.635
Tooth count (mean, SD)	5.3 (8.0)	6.7 (8.5)	0.013
Self-rated health (fair or poor) (%, *n*)	24.9 (49)	20.2 (105)	0.373

Values are medians and interquartile ranges unless stated otherwise. Meal provision included meals provided by the social services, voluntary services or other private help in the previous 4 weeks. Luncheon club comprises visits also in the previous 4 weeks. Swallowing problems included dry mouth and difficulty swallowing for other reasons. Protein intake distribution was calculated as SD/total protein intake with higher values reflecting more skewness of intakes across time intervals in the day

*aBW* adjusted body weight (kg), *BMI* body mass index, *CV* coefficient of variation, *hs*-*CRP* high sensitivity C-reactive protein, *SMMSE* standardised mini-mental state examination, *y* years

*Chi-squared test (*χ* ^2^) was used for categorical variables, independent *t* test for continuous normally distributed variables and Mann–Whitney *U* test for no protein intake difference for continuous non-normally distributed variables

^a^A protein intake < 0.8 g/kg aBW/day was considered low

^b^≥ 0.8 g/kg aBW/day was considered adequate. Body weight was adjusted to the nearest value to reflect a healthy BMI in older adults aged ≥ 71 years of 22–27 kg/m^2^ as described in Berner et al. [20]

### Contribution of food groups to protein intake

Those with adequate protein intake consumed more of almost every food group (weight in g). MMP contributed 6% more to protein intake (*p* < 0.001) while CCP (*p* = 0.009) and most other food groups contributed less to protein intake in the adequate protein intake group (≥ 0.8 g/kg aBW/day) than in the low protein group (< 0.8 g/kg aBW/day) (Table 2). Higher consumption (but also higher protein intake) of CCP, MMP and milk and milk products was associated with a lower likelihood of having low total protein intake (< 0.8 g/kg aBW/day) (Supplemental Table 1). However, when calculated as the percent contribution of food groups to total protein intake, only higher percent contribution of MMP to total protein intake was associated with reduced risk of low protein intake (OR 0.97, 95% CI 0.95, 1.00). On the other hand, higher percent contribution of CCP and non-alcoholic beverages to protein intake were associated with increased ORs of low protein intake. The models were adjusted for sex, energy intake, BMI and the other top protein-contributing food groups (MMP, CCP, milk and milk products and non-alcoholic beverages but not fish and fish dishes or egg and egg dishes as the percent of consumers was only 35% and 38%, respectively) (Supplemental Table 1).

**Table 2:** Consumption (g/day) and contribution of 15 food groups to protein intake (%) among consumers with low and adequate protein intakes

Food groups	Consumption weight (g/day)			Contribution to protein intake (%)		
	Low protein^a^	Adequate protein^a^	*p* value*	Low protein^a^	Adequate protein^a^	*p* value*
Meat and meat products	72.5 (43.4–110.4)	128.5 (82.5–187.3)	< 0.001	30.7 (19.6–40.5)	37.0 (23.8–47.7)	< 0.001
Cereals and cereal products	145.5 (105.3–203.8)	239.5 (164.0–334.5)	< 0.001	25.4 (19.2–32.2)	23.1 (17.5–30.0)	0.009
Fish and fish dishes	45.0 (23.8–60.0)	64.0 (42.0–103.0)	< 0.001	15.6 (10.4–21.5)	16.8 (10.2–25.8)	0.327
Milk and milk products	105.8 (50.0–195.8)	155.5 (75.8–245.6)	< 0.001	11.7 (6.2–20.6)	10.7 (6.4–17.4)	0.196
Eggs and egg dishes	30.0 (25.0–55.0)	30.0 (25.0–60.0)	0.108	10.4 (6.3–16.8)	7.0 (4.6–10.7)	< 0.001
Non-alcoholic beverages^b^	1100 (880–1415)	1265 (960–1573)	< 0.001	9.5 (6.0–12.2)	6.6 (4.2–8.9)	< 0.001
Nuts and seeds	13.8 (8.5–18.8)	15.5 (6.0–23.5)	0.760	5.4 (4.6–6.4)	3.6 (2.1–5.5)	0.071
Vegetables	89.0 (49.0–134.0)	114.5 (65.3–171.0)	< 0.001	4.5 (2.0–7.3)	3.5 (1.8–5.4)	0.002
Potatoes	84.3 (48.5–138.0)	110.5 (70.0–160.5)	< 0.001	4.0 (2.2–6.5)	3.1 (2.0–4.9)	0.002
Fruit	124.5 (85.3–197.8)	157.5 (84.8–247.3)	0.023	2.2 (1.2–3.5)	1.5 (0.8–2.6)	< 0.001
Savoury snacks	14.0 (8.4–14.0)	14.0 (7.0–17.1)	0.887	1.7 (1.1–2.7)	1.1 (0.7–1.5)	0.002
Miscellaneous	65.0 (20.0–150.0)	53.0 (22.0–141.0)	0.602	1.2 (0.3–5.3)	0.7 (0.2–2.9)	0.002
Sugar, preserves and confectionery	16.0 (10.0–32.6)	21.3 (11.4–39.0)	0.032	1.4 (0.0–2.0)	0.2 (0.0–1.2)	0.448
Alcoholic beverages	124.0 (50.0–445.5)	135.8 (53.8–288.5)	0.924	0.2 (0.0–2.3)	0.2 (0.0–0.7)	0.209
Oils and fat spreads	16.0 (9.0–24.0)	18.0 (12.0–28.0)	0.005	0.1 (0.1–0.3)	0.1 (0.1–0.3)	0.054

Values are medians and interquartile ranges

*aBW* adjusted body weight (kg)

*Mann–Whitney *U* test for no difference between low < 0.8 g/kg aBW/day) and adequate protein intake (≥ 0.8 g/kg aBW/day)

^a^Body weight was adjusted to reflect a healthy BMI in older adults of 22–27 kg/m^2^

^b^Includes tea/coffee with added milk

### Protein intake by eating occasion

**Fig. 1:**
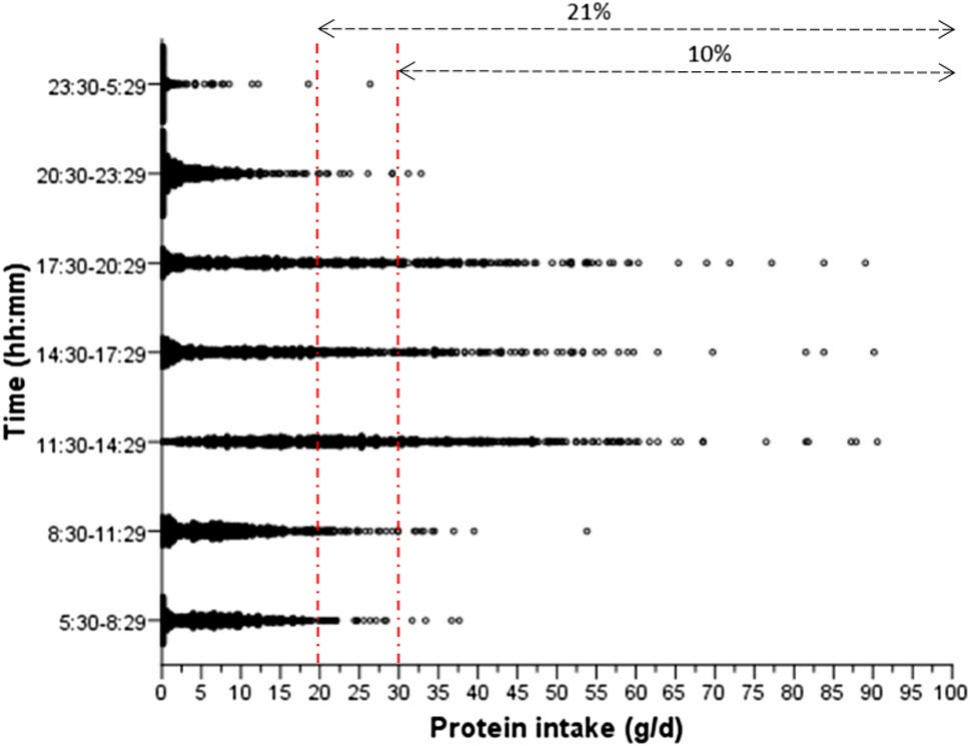
Protein intake (g/day) distribution per time category (meal) for individual participants (*n* = 722) in the Newcastle 85+ Study. The vertical-dashed lines represent the suggested protein amount of either 20 g/meal or 30 g/meal believed to be necessary for optimal protein synthesis. The arrows represent the % of all meals by all participants that meet that threshold. 75% (*n* = 542) of participants had a meal between 5:30 and 8:29, 89% (*n* = 643) from 8:30 to 11:29, 99% (*n* = 714) from 11:30 to 14:29, 90% (*n* = 646) from 14:30 to 17:29, 86% (*n* = 623) from 17:30 to 20:29, 73% (*n* = 112) from 20:30 to 23:29 and 16% (*n* = 112) from 23:30 to 5:29

Ninety-nine percent of participants in the analytic sample (*n* = 714) consumed a meal between 11:30 and 14:29 but only 16% (*n* = 112) consumed one between 23:30 and 5:29 (Fig. 1). Most of the protein intake [~ 35% or 20.4 g (12.8–30.4)] occurred during the “lunch” period (11:30–14:29) followed by the “dinner” period (17:30–20:29) [~ 21% or 12.0 g (4.2–24.3)]. The two morning eating occasions (5:30–8:29 and 8:30–11:29) combined, contributed to ~ 22% or 12.9 g (5.5–20.8) of the total protein intake. Those with protein intake ≥ 0.8 g/kg aBW/day had greater protein intake (g/day) in almost every eating occasion (time category) (Fig. 1 and Supplemental Table 2). However, percent contribution to total protein intake during the early part of the day (5:30–8:29 and 8:30–11:29) was higher in the low than in the adequate protein intake group (*p* = 0.002 and *p* = 0.004, respectively) (Table 3). This finding was confirmed by linear and binary logistic regression models adjusted for health and sociodemographic characteristics (*p* < 0.001) (Supplemental Table 3).

**Table 3:** Percent contribution of each eating occasion to protein intake by low or adequate protein intake

Time (hh:mm)	All	Low protein^a^	Adequate protein^a^	p*
5:30–8:29	11.0 (5.4–17.3)	13.2 (5.9–20.7)	10.2 (5.3–16.5)	0.002
8:30–11:29	10.5 (3.6–17.3)	12.9 (4.4–19.6)	9.3 (3.6–16.5)	0.004
11:30–14:29	34.6 (22.4–47.9)	35.0 (23.2–45.9)	34.3 (22.3–48.6)	0.566
14:30–17:29	17.1 (4.9–32.0)	16.9 (6.5–30.3)	17.3 (3.9–32.2)	0.832
17:30–20:29	20.8 (8.1–38.5)	19.9 (8.4–36.4)	21.9 (7.8–39.2)	0.504
20:30–23:29	4.4 (1.3–9.4)	4.3 (1.2–10.2)	4.5 (1.4–9.3)	0.738
23:30–5:29	0.0 (0.0–2.2)	0.0 (0.0–2.2)	0.0 (0.0–1.6)	0.415

Values are medians and interquartile ranges

*aBW* adjusted body weight (kg), *hh* hours, *mm* minutes

*Mann–Whitney *U* test for no difference between low (< 0.8 g/kg aBW/day) and adequate protein intake (≥ 0.8 g/kg aBW/day) categories

^a^Body weight was adjusted to reflect a healthy body mass index in older adults of 22–27 kg/m^2^

Twenty-one percent and 10% of the eating occasions had more than 20 and 30 g of protein, respectively. These eating occasions occurred mostly during the “lunch” (11:30–14:29) and “dinner” period (17:30–20:29) or in between (14:30 and 17:29). For example, half (52%) of every “lunch” eating occasion but only 4% (5:30–8:29) and 6% (8:30–11:29) of “breakfast” eating occasions had more than 20 g of protein [25] (Fig. 1). Consumption of at least 20 g of protein within any one eating occasion was predictive of adequate protein intake after adjustment for sex, energy intake and BMI (OR 0.07, 95% CI 0.04, 0.13). There were insufficient numbers of participants (*n* = 15) who had both low total protein intakes (< 0.8 g/kg aBW/day) and an eating occasion with ≥ 30 g of protein to replicate the analysis for this cutoff.

### Predictors of protein intake

Using backward stepwise multivariate linear regression modelling with a 90% significance level, significant predictors of higher protein intake adjusted for key covariates being female (*β* = 0.087 ± 0.026, *p* < 0.001), having higher energy intake (MJ/day) (*β* = 0.109 ± 0.006, *p* < 0.001) and higher tooth count (*β* = 0.003 ± 0.001, *p* = 0.047). Significant predictors of lower protein intake included drinking alcohol (*β* = − 0.049 ± 0.026, *p* = 0.067) and having swallowing problems (*β* = − 0.040 ± 0.023, *p* = 0.077) (Table 4).

**Table 4:** Factors associated with protein intake per adjusted body weight (g/kg aBW/day)

	Non-standardised *β*	SE	95% CI	*p*
All (adjusted *R* ^2^ = 0.443)				
Constant	0.208	0.056	0.098, 0.319	< 0.001
Sex (men)	(Ref.)			
(Women)	0.087	0.026	0.036, 0.138	0.001
Energy intake (MJ/day)	0.109	0.006	0.098, 0.121	< 0.001
Alcohol drinker (no)	(Ref.)			
(Yes)	−0.049	0.026	−0.101, 0.003	0.067
Tooth count	0.003	0.001	0.00, 0.005	0.047
Swallowing problems (no)	(Ref.)			
(Yes)	−0.040	0.023	−0.085, 0.004	0.077
Women (adjusted *R* ^2^ = 0.437)				
Constant	0.187	0.057	0.075, 0.298	0.001
Energy intake (MJ/day)	0.119	0.009	0.102, 0.136	< 0.001
Tooth count	0.005	0.002	0.001, 0.008	0.007
Men (adjusted *R* ^2^ = 0.425)				
Constant	0.302	0.076	0.168, 0.468	< 0.001
Energy intake (MJ/day)	0.104	0.008	0.088, 0.120	< 0.001
Alcohol drinker (no)	(Ref.)			
(Yes)	−0.087	0.050	−0.185, 0.010	0.080

Sex, disease count, years of full-time education, past occupation (NS-SEC), living alone, energy and alcohol intake, smoking, physical activity, self-rated health, diet change, Geriatric Depression Scale, standardized mini-mental state examination, disease count, number of medications, renal impairment, tooth count, swallowing problems, meal provision, luncheon club attendance, ability to go shopping and cook a hot meal were entered into the backward stepwise multivariate linear regression. Swallowing problems included dry mouth and difficulty swallowing for other reasons

*CI* confidence interval, *SE* standard error

## Discussion

Lower protein intake defined by different cutoffs has been associated with loss of muscle mass and strength, increased disability count, loss of independence and mortality in several cohorts of older adults [26–29]. More than one quarter of the Newcastle 85+ Study had low protein intakes defined as protein intake < 0.8 g/kg aBW/day. Higher contribution of MMP to total protein intake reduced the risk of low protein intake, while higher percent contribution of CCP and non-alcoholic beverages was associated with increased ORs of having low protein intake. Those who consumed more of their total protein intake during the morning eating occasions were more likely to be in the low than in the adequate total protein intake group (≥ 0.8 g/kg aBW/day). Being a woman, having higher energy intake and higher tooth count was associated with adequate protein intake in adjusted models.

We expressed protein intake per adjusted body weight to estimate the prevalence of low protein intake (< 0.8 g/kg aBW) as used in the National Health and Nutrition Examination Survey (NHANES) 2005–2006 of over 1700 participants aged 51 and over [20]. The use of actual or adjusted body weight (adjusted to reflect a healthy BMI range of 22–27 associated with favourable health outcomes in those ≥ 71 years) affected the estimate for the prevalence of protein inadequacy by 1% (*n* = 10) and mostly in women. Although the debate about the best way to calculate the protein requirements and the prevalence of low protein intake in older adults is still ongoing, using adjusted body weight may be more sensible to detect the population at risk. In this study the median protein intake was 61.3 (IQR 48.9–75.7) g/day (15.7% of energy intake) [21] equivalent to 0.97 (0.77–1.20) g/kg aBW/day and 28% of the participants had protein intakes below 0.8 g/kg aBW/day. Mean/median protein intakes of European older adults ≥ 80 years range from 60.9 to 89.7 g/day [30] or 0.94–1.38 g/unadjusted kg BW/day (assuming a mean body weight of 65 kg), which is above the current RDA [31].

Protein intake at the current RDA of 0.8 g/kg BW/day may maintain positive nitrogen balance for a short period only and lead to loss of muscle mass over time in older adults [4]. Others suggested that older adults had protein needs greater than the current RDA [10, 11, 32]. The PROT-AGE study group and later the European Society for Clinical Nutrition and Metabolism (ESPEN) expert group recommended that the protein RDA for older adults should be increased to 1–1.2 g/kg/day [10, 11]. The Nordic Nutrition Recommendations suggested an even higher protein RDA of 1.2–1.4 g/kg BW/day [33].

MMP was the biggest contributor to total protein intake, followed by CCP, fish and fish dishes, and milk and milk products [34], similar to the top protein intake contributors in older adults (≥ 65 years) of the NDNS rolling programme years 5–6 [35]. Participants where MMP contributed more to their protein intakes were less likely to be in the low protein intake group after adjustment for sex, energy intake, BMI and other top food protein contributors (CCP, milk and milk products, non-alcoholic beverages). CCP were also ubiquitously consumed in the Newcastle 85+ Study (data not shown) and assume a more important role as a contributor to protein intake than in younger populations. Therefore, dietary interventions to increase protein intake in the very old focusing on CCP would be promising. Those with more skewed protein distribution had higher protein intake in unadjusted models. However, additional adjustment for percent contribution of MMP to protein intake reduced the association to non-significant, suggesting that considerable intakes of MMP during one or two meals were the reason why skewness was higher in those with better total protein intake. An intake of > 20 g per meal occasion has been suggested as necessary to stimulate muscle protein synthesis in older adults [25], although the results from intervention studies investigating the benefits of even versus skewed/pulse protein intake for muscle synthesis have been mixed [36–38]. The Quebec Longitudinal Study on Nutrition and Ageing (NuAge) study, which included more than 700 older adults aged 67–84 years, found that participants with more even protein distribution throughout the day had higher lean mass and appendicular lean mass than those with a more skewed protein distribution during the 2-year follow-up [37]. However, a randomized controlled trial of 66 malnourished or at-risk hospitalised older adults (> 70 years) found that those who had a skewed protein intake (72% of total protein intake was consumed in a single meal) gained significantly more lean mass over 6 weeks compared to those that had a more even distribution of protein intake in four meals across the day [38].

In this study, morning meals contributed more to total protein intake in the low than in the adequate protein intake group (but not more than e.g. “lunch” eating occasions). This is in agreement with others [39] which postulated that a higher contribution of morning meals to protein intake might produce greater satiety than later in the day. In fact, this observation does not seem limited to protein intake as this has also been observed with carbohydrate and fat [39].

In the present study, being a woman, having higher energy intake and higher tooth count was associated with higher protein intake (adjusted models). A recent systematic review examining the determinants of malnutrition (different outcomes used such as involuntary weight loss, low weight, low energy intake and/or appetite loss) in community-dwelling older adults (≥ 60 years) identified more than 100 potential-associated factors [40]. From these, poor appetite, diabetes diagnosis, edentulousness, more recent hospitalisation and poor self-reported health were associated with low protein–energy intake [40]. Different outcomes, different model adjustments, age of participants, and, above all, the multifactorial nature of malnutrition might explain the discrepancies between the findings.

The strengths and uniqueness of the Newcastle 85+ Study lie with the large number of very old included (sociodemographically representative of the UK [13]), the multidimensional health data collected and the assessment of dietary intake using a validated approach [17, 21, 41]. Assessing dietary intake in the very old poses challenges (described in detail by Adamson et al. [17]), and potential misreporting is one of them (estimated to be 26% in the Newcastle 85+ Study [21] with 22% underreporting). However, because protein-rich foods are less commonly underreported (unlike snacks and sweets) [42] and the 24 h-MPRs included several prompts, it is unlikely that protein intakes were underestimated. For fish, there may be a slight possibility of underreporting because the fish consumption in the UK is traditionally higher on Fridays [43], and the two 24 h-MPRs were not conducted during the weekend, (therefore, Fridays and Saturdays were not included) [41]. Weight and appetite loss are very important predictors of reduced food intake and malnutrition [40, 44]. Inclusion of these questions in our multidimensional health assessment would be invaluable to this study and strengthen our conclusions. Participants were weighed at subsequent follow-up phases. Fourteen percent (*n* = 76) of our sample had ≥ 5% of weight loss from baseline to phase 2 (after 18 months) and 40% (*n* = 159) had ≥ 5% of weight loss from baseline to phase 3 (after 36 months). However, as with any prospective observational study of the very old, the attrition rates were high. Therefore, analyses could not be stratified for weight-loss alone or in combination with low protein intake (< 0.8 g/kg aBW/day) due to low numbers in each category (e.g. low protein intake and weight loss). Another limitation in relation to protein intake from the 15 food groups used is that these were not disaggregated, and therefore, not only included single items (e.g. beef steak) but also composite dishes (e.g. shepherd’s pie, minced beef with potato topping).

## Conclusion

This study provides evidence that low protein intake is prevalent in the very old. In addition, we provide information on protein intake patterns and food group contributors to protein intake which could be helpful when considering interventions to improve protein intake in this fast-growing population group.

## Electronic supplementary material

Below is the link to the electronic supplementary material.

Supplementary material 1 (PDF 225 kb)

## Abbreviations

CCP: Cereals and cereal products
MMP: Meat and meat products
RDA: Recommended dietary allowance
