# Supplementary material for: Prevalence and determinants of low protein intake in very old adults: insights from the Newcastle 85+ Study
Source: Eur J Nutr. 2017 Sep 25;57(8):2713–22. doi: 10.1007/s00394-017-1537-5 (PMC6267410; doi:10.1007/s00394-017-1537-5)
Supplement: Supplementary file 1 — Supplementary material 1 (PDF 225 kb) [file 394_2017_1537_MOESM1_ESM.pdf]

Prevalence and predictors of low protein intake in very old adults: Insights from the Newcastle 85+ Study

Nuno Mendonça[1-3*], Antoneta Granic[2,4,5], John C. Mathers[2,3,6], Tom R. Hill[1-3], Mario Siervo[2,3,6], Ashley J. Adamson[2,3,7], Carol Jagger[2,7]

[1] School of Agriculture Food and Rural Development, Newcastle University, Newcastle upon Tyne, NE1 7RU, UK.

[2] Newcastle University Institute for Ageing, Newcastle University, NE2 4AX, UK.

[3] Human Nutrition Research Centre, Newcastle University, NE2 4HH, UK.

[4] Institute of Neuroscience, Newcastle University, NE2 4HH, UK.

[5] NIHR Newcastle Biomedical Research Centre in Ageing and Chronic Disease, Newcastle University and Newcastle upon Tyne NHS Foundation Trust, NE4 5PL, UK.

[6] Institute of Cellular Medicine, Newcastle University, NE4 5PL, UK.

[7] Institute of Health and Society, Newcastle University, NE4 5PL, UK.

*****Corresponding author: Nuno Mendonça, email n.m.p.mendonca@newcastle.ac.uk

Supplemental methods

Binary logistic regression models were used to estimate the ORs of having low protein intake (<0.8 g/kg aBW/day) by top protein contributing food groups. Models were adjusted for sex, energy intake, BMI and the remaining top protein contributing food groups. Fish and fish dishes, and eggs and egg dishes were a top contributor to protein intake among consumers but these were only consumed by 35 and 38% of the analytical sample, respectively, hence these were not included. Linear and binary logistic regression models were also used to estimate the βs and ORs of total protein intake (g/d), total protein intake by adjusted body weight (g/kg aBW/d) and low protein intake (<0.8 g/kg aBW/d), respectively, by percentage contribution of morning eating occasions to total protein intake. Model 1 is unadjusted. Model 2 is further adjusted for gender, total energy and BMI. Model 3 is further adjusted for previous occupation (NS-SEC), living alone, physical activity, alcohol intake, number of medications used, swallowing problems, tooth count, disease count and renal impairment. Morning eating occasions (breakfast) were broadly defined as any meal eaten from 5h30-11h30 a.m. Statistical analysis was conducted using the IBM statistical tool SPSS v22.0. $p<0.05$ was considered statistically significant unless otherwise mentioned.

Supplemental Table 1. Binary logistic regression models for low protein intake (<0.8 g/kg aBW/day) by top protein contributing food groups.

**Food groups**	Consumption weight (100 g/d)		Contribution to protein intake (%)	
	**OR** (95% CI)	*p*	**OR** (95% CI)	*p*
Meat and Meat Products	0.28 (0.17, 0.47)	<0.001	0.97 (0.95, 1.00)	0.020
Cereals and Cereal Products	0.58 (0.41, 0.82)	0.002	1.06 (1.03, 1.10)	<0.001
Milk and Milk Products	0.62 (0.48, 0.80)	<0.001	0.99 (0.96, 1.02)	0.627
Non-Alcoholic Beverages[a]	0.94 (0.88, 1.01)	0.089	1.11 (1.04, 1.20)	0.004

[a] Includes Tea/Coffee with added milk. Models are adjusted for sex, energy intake, body mass index and the other top protein contributing food groups. Fish and fish dishes, and eggs and egg dishes were a top contributor to protein intake among consumers but these were only consumed by 35 and 38% of the analytical sample, respectively. OR, odds ratio.

Supplemental Table 2. Protein intake (g/d) by time category and by low protein intake cut-offs.

Time (hh:mm)	**All**	**<0.8 g/kg aBW/day**[a]	**≥0.8 g/kg aBW/day**[a]	*p*[*]
5:30-8:29	6.6 (3.2-10.4)	5.3 (2.5-9.0)	7.3 (3.6-11.4)	0.002
8:30-11:29	6.3 (2.3-10.4)	5.4 (1.8-8.6)	6.5 (2.5-11.0)	0.006
11:30-14:29	20.5 (12.9-30.5)	13.8 (8.4-20.3)	23.2 (15.1-35.2)	<0.001
14:30-17:29	10.0 (2.7-19.3)	7.4 (2.6-12.6)	11.9 (3.1-22.2)	<0.001
17:30-20:29	12.3 (4.3-24.3)	8.1 (3.1-14.5)	14.0 (5.0-27.9)	<0.001
20:30-23:29	2.6 (0.8-6.0)	1.7 (0.5-4.1)	3.1 (0.9-6.6)	<0.001
23:30-5:29	0.0 (0.0-1.2)	0.0 (0.0-2.2)	0.0 (0.0-1.0)	0.632

[a] Body weight was adjusted to reflect a healthy BMI in older adults of 22-27 kg/m2. [*] Mann-Whitney U test for no difference between low and high protein intake categories. Values are medians and interquartile ranges. aBW, adjusted body weight (kg); hh, hours; mm, minutes.

Supplemental Table 3. Associations between the percentage contribution of morning eating occasions to total protein intake and total protein intake, protein intake by adjusted body weight and low protein intake.

	**Total protein intake (g/day)**		**Protein intake by adjusted body weight (g/kg aBW/day[a])**		**Low protein intake (<0.8 g/kg aBW/day[a])**	
	β (SE)	*p*	β (SE)	*p*	OR (95% CI)	*p*
Model 1	-0.50 (0.08)	<0.001	-0.008 (0.001)	<0.001	1.05 (1.03-1.06)	<0.001
Model 2	-0.34 (0.06)	<0.001	-0.005 (0.001)	<0.001	1.05 (1.03-1.08)	<0.001
Model 3	-0.53 (0.05)	<0.001	-0.006 (0.001)	<0.001	1.07 (1.04-1.11)	<0.001

[a] Body weight was adjusted to reflect a healthy BMI in older adults of 22-27 kg/m2. Model 1 is unadjusted. Model 2 is further adjusted for gender, total energy and BMI. Model 3 is further adjusted for previous occupation (NS-SEC), living alone, physical activity, alcohol intake, number of medications used, swallowing problems, tooth count, disease count and renal impairment. Morning eating occasions (breakfast) were broadly defined as any meal eaten from 5h30-11h30 a.m. BMI, body mass index; CI, confidence interval; OR, odds ratio; SE, standard error.

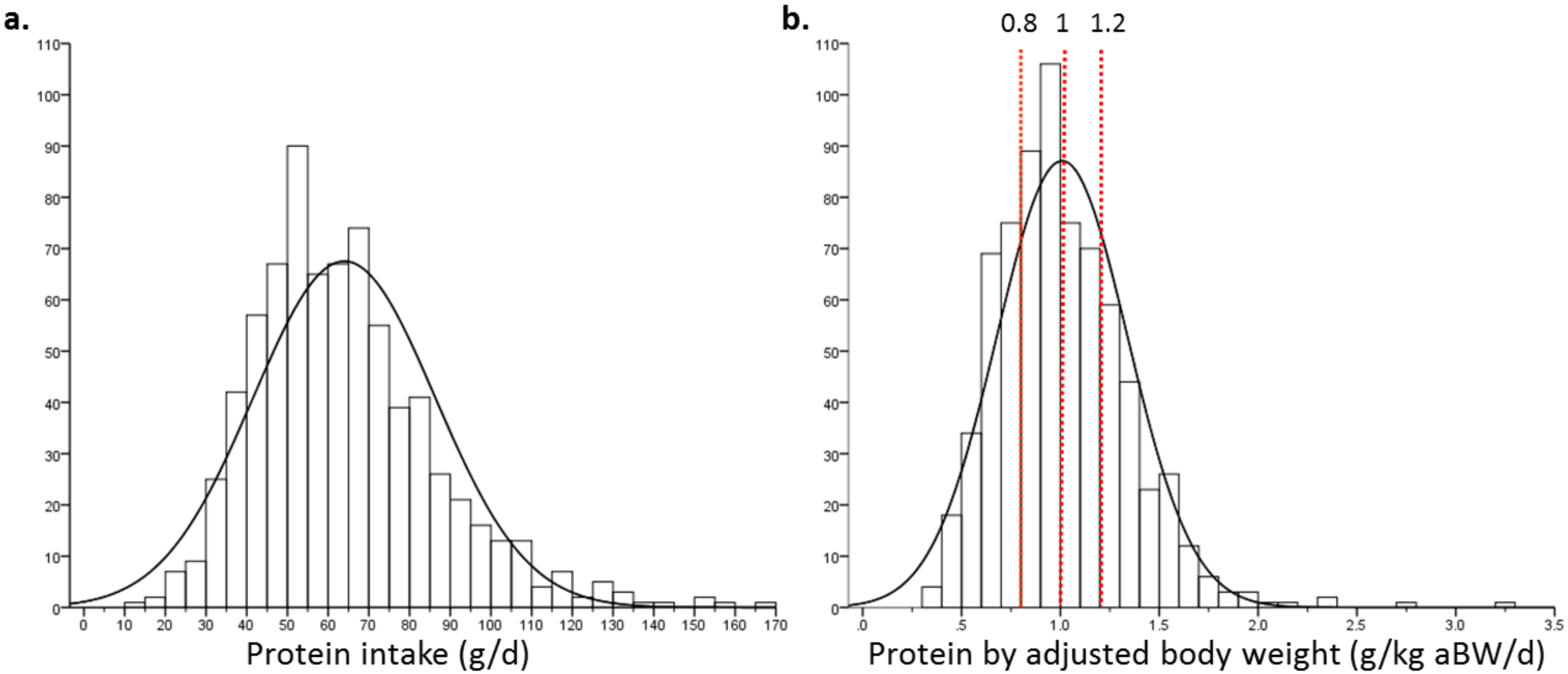

Supplemental Figure 1. Distribution of **a.** protein intake (g/d) and **b.** protein intake by adjusted body weight (g/kg aBW/d). Body weight was adjusted to reflect a healthy body mass index in older adults of 22-27 kg/m^2. 28% (n=199) of participants had protein intakes <0.8 g/kg aBW/d, 54% (n=390) <1.0 g/kg aBW/d and 75% (n=539) <1.2 g/kg aBW/d. aBW, adjusted body weight.
